# Underutilized crops for diverse, resilient and healthy agri-food systems: a systematic review of sub-Saharan Africa

**DOI:** 10.3389/fsufs.2024.1498402

**Published:** 2024-11-28

**Authors:** Mendy Ndlovu, Pauline Scheelbeek, Mjabuliseni Ngidi, Tafadzwanashe Mabhaudhi

**Affiliations:** 1 Centre for Transformative Agricultural and Food Systems, School of Agricultural, Earth and Environmental Sciences, University of KwaZulu-Natal, Pietermaritzburg, South Africa; 2 Discipline of Agrometeorology, School of Agricultural, Earth and Environmental Sciences, University of KwaZulu-Natal, Pietermaritzburg, South Africa; 3 Centre on Climate Change and Planetary Health, London School of Hygiene and Tropical Medicine, London, United Kingdom; 4 Discipline of Agricultural Extension and Rural Resource Management, School of Agricultural, Earth and Environmental Sciences, College of Agriculture, Engineering and Science, University of KwaZulu-Natal, Pietermaritzburg, South Africa

**Keywords:** future crops, nutrition, sustainable diets, food sovereignty, agrobiodiversity, climate change adaptation

## Abstract

Sub-Saharan Africa (SSA) faces increasing water scarcity, food and nutrition insecurity, poverty and inequality under climate change. Under these circumstances, promoting locally adapted and nutrient-dense crops is touted as a plausible climate adaptation strategy. We reviewed the utility of neglected and underutilized crop species (NUS) as a climate change adaptation strategy to diversify local food systems and diets and improve nutritional health and environmental outcomes in SSA. We conducted a systematic literature review using Web of Science and Scopus research databases. Of the 1,545 studies retrieved, 75 were included following a multi-level screening process on Covidence guided by the Preferred Reporting Items for Systematic reviews and Meta-Analyses (PRISMA) guidelines. The review consolidates fragmented knowledge on the application of NUS in different contexts. Despite growing interest, NUS remain gendered and stigmatized crops, marginalized and fragmented in research, development, and marketing efforts and lack explicit support from policy and decision-makers. Despite rhetoric purporting to support them, there is a worrying rise in policies and regulations that inadvertently hinder the development of these crops and reinforce dependence on a narrow basket of crops for food and nutrition security, undermining food sovereignty. Some NUS have received increasing recognition for their potential in the past decade. However, this is neither universal nor systematic, which makes scaling up necessary but challenging. Consequently, progress in mainstreaming NUS in local food systems continues to lag. Despite these challenges, NUS remain sub-Saharan Africa’s better-bet option for diversifying food systems and transitioning them to be equitable, inclusive, resilient and healthy; hence, NUS provide positive outcomes for people and the planet under climate change.

## 1 Introduction

Underutilized crops, also referred to as orphan, indigenous, traditional crops, or neglected, underutilized species (NUS) (Mabhaudhi et al., 2017a), are local crop varieties that communities have utilized for generations. However, they have been rendered neglected due to the promotion and dominance of a few major/staple crops and an industrial monoculture-centered agri-food system (Mabhaudhi et al., 2017a; Sirany et al., 2022; Popoola et al., 2022; Andani et al., 2022; Abberton et al., 2022; Feleke and Tekalign, 2022; Mudau et al., 2022). Consequently, the value chains of NUS in Sub-Saharan Africa (SSA) are currently poorly developed, and smallholder farmers, especially women, in marginalized settings are currently the main conservators of these crops (Mabhaudhi et al., 2017a; Bilali, 2020; Hendre et al., 2019; Chivenge et al., 2015; Mabhaudhi et al., 2018b, 2016; Kodzwa et al., 2023). As a result, some NUS are at risk of abandonment (Sidibé et al., 2020).

NUS are typically more resilient to localized environmental stress, including drought, salinity, and extreme heat, generally have richer nutritional composition and require fewer resources than their “mainstream” counterparts (van Zonneveld et al., 2023; Nhamo et al., 2022; Mabhaudhi et al., 2018a; Chase et al., 2023). SSA has a huge, neglected potential for increasing production, utilization, and niche-specific competitiveness of and commercializing underutilized crops. Inclusion of these crops can improve the resilience, sustainability and inclusiveness of current agricultural and food systems, address hunger and food and nutrition insecurity challenges and support local developmental goals and livelihood strategies (Mabhaudhi et al., 2017a; Mugiyo et al., 2021). Therefore, the continued underutilization of NUS could be a major missed opportunity to contribute to SSA’s agri-food systems transformation and human and environmental wellbeing.

Food systems, human wellbeing, and planetary health are interlinked (World Health Organization, 2021; Richardson et al., 2023). Poor agri-food systems development and lack of diversity contribute to poor dietary composition, malnutrition, and negative health and food security outcomes (van Zonneveld et al., 2023; Hart et al., 2021; Mashamaite et al., 2022). The number of hungry people has been increasing globally since 2014, particularly in SSA, where 31 of the top 36 countries have the highest hidden hunger index, indicating a lack of access to essential nutrients (Hendre et al., 2019; Mekonnen et al., 2022; Bokelmann et al., 2022; van Zonneveld et al., 2021). This is a reversal of the progress made due to food-security-specific interventions, such as introducing the existing agri-food systems, which managed to reduce global hunger levels between 1990 and 2014 (Food Agriculture Organization, 2015). However, micronutrient deficiencies of iron, vitamin A, and iodine remain widespread in SSA, affecting the health and wellbeing of many of the population, particularly women and children (Mabhaudhi et al., 2017b; Jiri et al., 2017). Further, suppose no necessary adaptation strategies are employed urgently, by the year 2050, the world is expected to see a 20% increase in hunger, and close to 65% of those experiencing hunger will reside in SSA (Jiri et al., 2017).

The existing agri-food system challenges in SSA stem from the region’s dependence on agriculture (mainly rainfed), alongside inherently low adaptive capacity, widespread poverty with about 40% of the population living below the international poverty line in 2019, high socio-economic inequality rates, failing governance and a fast-growing population (Sirany et al., 2022; Popoola et al., 2022; Chivenge et al., 2015; Mekonnen et al., 2022; Food Agriculture Organization, 2015). Often, SSA countries rely extensively on climate-sensitive, misaligned, outdated and vulnerable mainstream agri-food systems, leading to food production insecurity, low yields, limited availability of food, poverty, and increasing food and nutrition insecurity as the climate changes (Sirany et al., 2022; Popoola et al., 2022; Andani et al., 2022; Bilali, 2020; Mekonnen et al., 2022; Akinola et al., 2020; Mubaiwa et al., 2018). These challenges coexist with the lack of appropriate-scale food and agricultural interventions, technologies and infrastructure to inform the region’s development and environmental, socioeconomic and policy-related barriers (Sirany et al., 2022; Mugiyo et al., 2021; Jiri et al., 2017; Akinola et al., 2020).

While there have been major strides made in improving agricultural productivity in SSA, the current agri-food systems in SSA are not resilient. The COVID-19 pandemic, alongside widespread global conflicts, climate crises and rising levels of malnutrition, have exposed its vulnerability to external shocks (Mashamaite et al., 2022; Nkwonta et al., 2023; Veldsman et al., 2023; Zhang and Dannenberg, 2022). These agri-food systems lack inclusion, equality and equity and embrace the production of a select few high-yielding, climate-sensitive mainstream crops with limited nutritional value, lacking in micronutrients and healthy proteins and have low dietary composition and diversity (Hendre et al., 2019; Mabhaudhi et al., 2018b; Mekonnen et al., 2022; Akinola et al., 2020; Agulanna, 2020; Cheng et al., 2017; Leakey et al., 2021). By design, the existing agri-food systems suppress crucial local agricultural systems that have previously informed diverse agri-food systems and could still contribute to sustainable and inclusive agri-food systems (Nhamo et al., 2022; Zhang and Dannenberg, 2022; Koch et al., 2021). These agri-food systems were shaped by the Green Revolution’s framework, colonialism, and the widespread capitalization of pre-existing agri-food systems (Nkwonta et al., 2023; Zhang and Dannenberg, 2022; Agulanna, 2020). This approach inadvertently undermines the importance of indigenous knowledge and farming systems and locally adapted agri-food systems (Nkwonta et al., 2023; Zhang and Dannenberg, 2022; Koch et al., 2021; Chemura et al., 2022). In its various versions, despite its successes, the framework failed to acknowledge and incorporate context-specific natural capital and localized systems to inform ethical, feasible and appropriate transformation (Zhang and Dannenberg, 2022; Leakey et al., 2021).

Uncertain environmental conditions, including climate change, further exacerbate these challenges. Sub-Saharan Africa mostly has a drought-prone, semi-arid climate and highly variable hydrometeorological regimes (Mabhaudhi et al., 2017a; Bilali, 2020; Paliwal et al., 2020). The region is among the global climate change hotspots due to repeated exposure to extreme weather events and underlying factors known to contribute to vulnerability (Sirany et al., 2022; Mekonnen et al., 2022). Climate change, higher frequency and intensity of weather extremes and hydrometeorological challenges, pests and diseases, soil degradation and erosion form additional challenges for agricultural production and productivity, leading to further declines in yields and increased crop failures across SSA (Sirany et al., 2022; Richardson et al., 2023; Mekonnen et al., 2022).

The central role of agriculture as a determinant of food and nutrition security and diet and health outcomes cannot be questioned, and innovative measures must be taken to improve agricultural systems to be food- and nutrition-sensitive in SSA. The different pathways to which NUS have been applied or promoted to contribute toward addressing such challenges in SSA have not been comprehensively examined, particularly in SSA in the context of agri-food systems development under climate change. In this review, we examine the state of knowledge on the application, utilization and existing constraints around adopting NUS to promote diverse, sustainable, and resilient agri-food systems and food sovereignty in sub-Saharan Africa under climate change. Understanding the utility and importance of NUS crops will help guide agricultural policy pathways in the region toward productive and resilient food and nutrition security-centered approaches. While this review advocates for food systems diversification through incorporating NUS, it does not advocate for the replacement of current major crops but argues that there is value to be gained from broadening the current basket of crops.

## 2 Methods

### 2.1 Defining NUS

In this systematic review, we collectively refer to the crops being spotlighted as Neglected and Underutilized crops (NUS), as explained by Chivenge et al. (2015) and Akinola et al. (2020). In short, this includes generational and historically important crops that have been a crucial part of native or local smallholder agricultural systems and played a role in contributing to agrobiodiversity, socio-cultural outcomes, food and nutrition security and food sovereignty at different localities during adversities. Most of these crops were primarily side-lined following the introduction of current “major crops” focused agri-food systems.

### 2.2 Identification of studies: inclusion and exclusion criteria

The search strategy used combined three main concepts or specific key terms: indigenous OR neglected OR traditional OR orphan OR native OR underutili$ed crops AND climat∗ change OR climat∗ variability OR global warming OR extreme weather OR greenhouse effect AND Sub-Saharan Africa OR SSA OR Sub Saharan Africa, including Comoros, Madagascar, Mauritius, and Seychelles island states in the strings tailored to conduct a search within Title-Abstract-Keyword on the Scopus database and inclusive Topic search (TS) on Web of Science (see Text 1 on the Supplementary material). All qualifying peer-reviewed research published in English, between January 2000 and December 2023 was considered and included if qualifying. The overall in-and-exclusion criteria used in the review are presented in Table 1. Specific authors were contacted for all full texts that we could not retrieve.

### 2.3 Data extraction, synthesis and analysis

**Figure 1:**
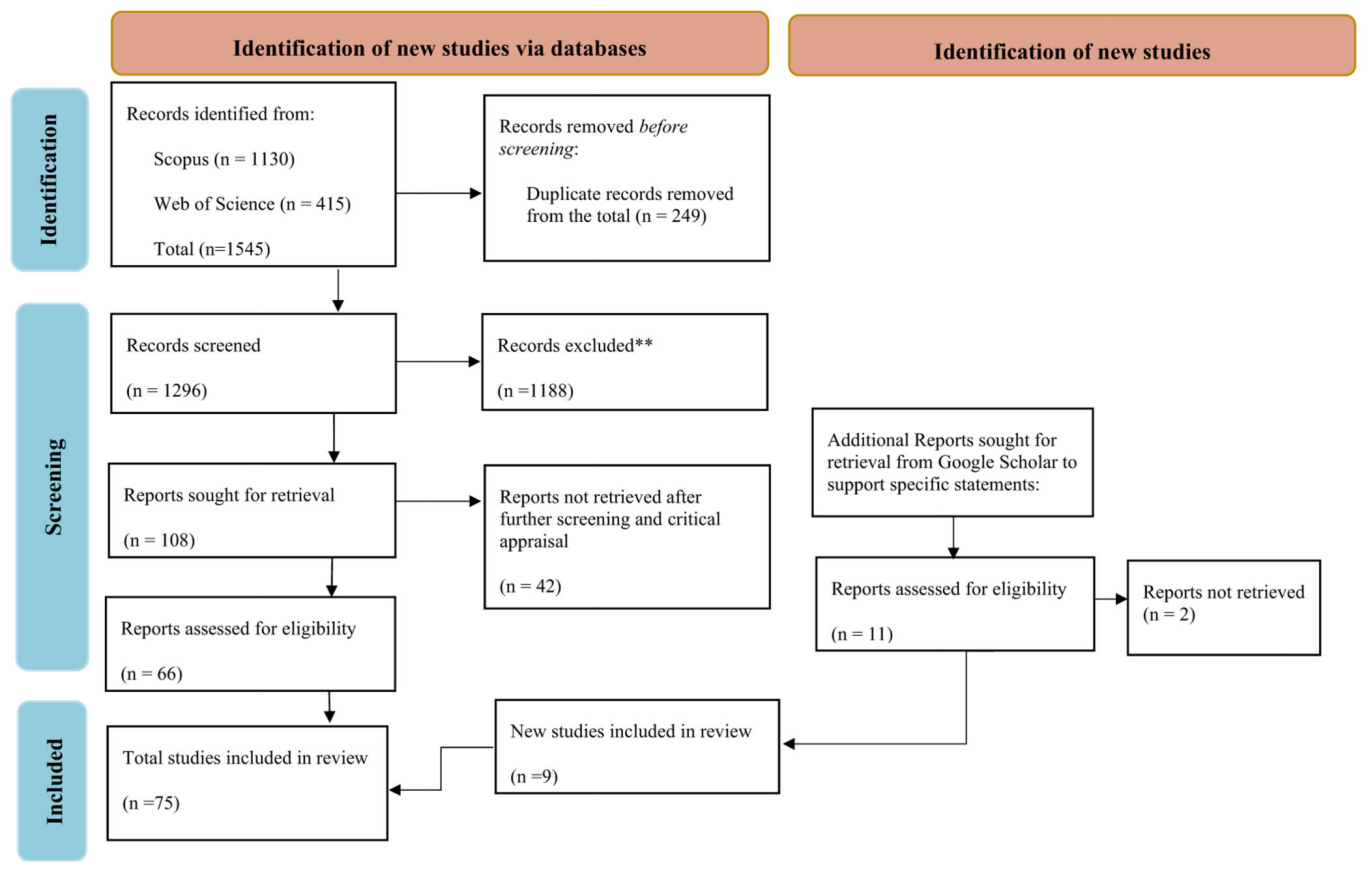
PRISMA flow chart detailing the study selection protocol adopted in this systematic review.

All references from Scopus or Web of Science research databases (1,545) were exported as RIS files to import into Covidence for deduplication and screening following the Preferred Reporting Items for Systematic Reviews and Meta-Analyses (PRISMA) guidelines (Figure 1). Screening on Covidence was performed by two authors to manage bias, while one author completed full-text screening, critical appraisal, and data extraction using Microsoft Excel and a form generated using Microsoft Word. All authors reviewed and validated critical appraisal, data extraction and analysis. The year of publication, country of focus within the region, reported use and potential application of NUS, NUS and their prioritization in research, research outcomes and gaps in NUS prioritization, and barriers to NUS adoption were extracted as emerging themes and mutually inclusive categories identified, and studies were grouped according to these themes. The secondary review of extracted data and the data analysis protocol from all included studies was done by all authors. Data analysis and initial reporting were done by a single author and shared with the team for collaborative data cleaning, refining, validation, insights and examination for potential biases and human errors.

### 2.4 Thematic and statistical analyses

Because of the wide variety of available literature on the subject matter, we adopted both narrative and quantitative approaches for data analysis and synthesis. We performed a thematic coding and analysis for all studies included by categorizing studies into mutually inclusive categories (articles with similar study outcomes, reported use/potential use of NUS for health and wellbeing outcomes, and environmental and socio-cultural outcomes). The included articles were independently coded using a descriptive/thematic/analytical analysis approach by one researcher and independently reviewed by three researchers as secondary coding. Information extracted for quantitative analysis included year of publication, prioritized NUS crops in research and prioritized outcomes for NUS application in research, allowing for a synthesis of priorities, reported uses, research focus, and potential of NUS to strengthen climate change adaptation strategies at different levels for improved agricultural and food production sustainability, and food, nutrition and health outcomes according to existing research. The statistical analysis of this data was conducted using percentage calculations of extracted information, for example, percentages of publications prioritizing NUS for a specific outcome, such as enhancing food security, to the entire database of included studies.

### 2.5 Critical appraisal

The set research objective, inclusion and exclusion criteria and the PRISMA systematic review guidelines guided evidence synthesis for the current study. For the final screening stage, we performed content analyses. We adapted the Critical Appraisal Skills Programme’s checklist (see Supplementary material, Table 2) using only questions that applied to this review to qualify articles that formed a part of this study. We considered specific questions to exclude poor-quality studies instead of conducting a full critical appraisal, which doesn’t apply to this type of study. Responses to our adapted/modified critical appraisal questions informed the final inclusion or elimination of full-text articles that had passed through different PRISMA-guided screening phases. One author independently appraised each article, and all authors approved the appraisal. We excluded articles that had:

- unclear or absent methodology sections,
- an unclear or absent synthesis of findings,
- did not have formulated research questions.

## 3 Results

### 3.1 Literature search

**Figure 2:**
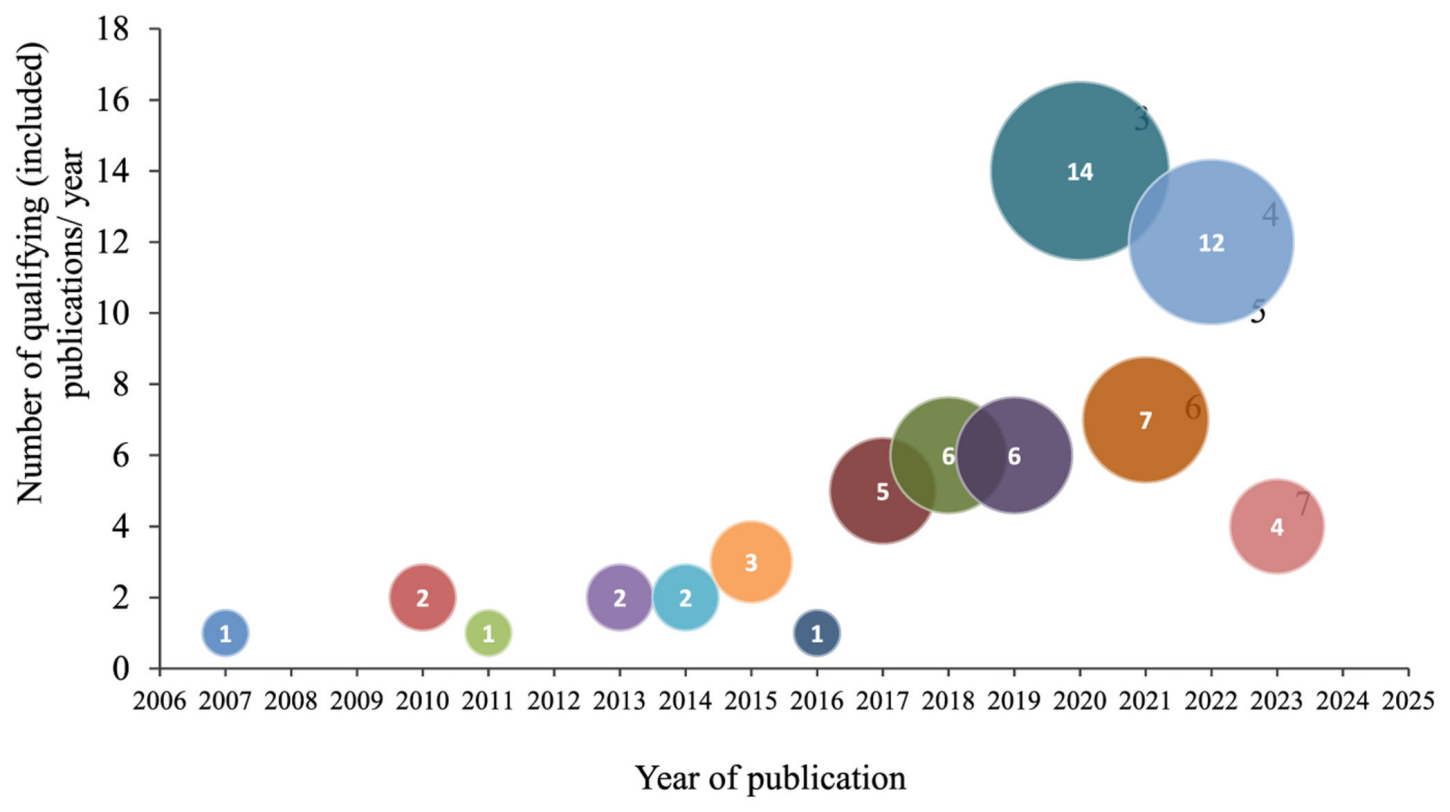
The number of publications on NUS over the years. Studies included in this research were published between January 2000 and December 2023.

**Figure 3:**
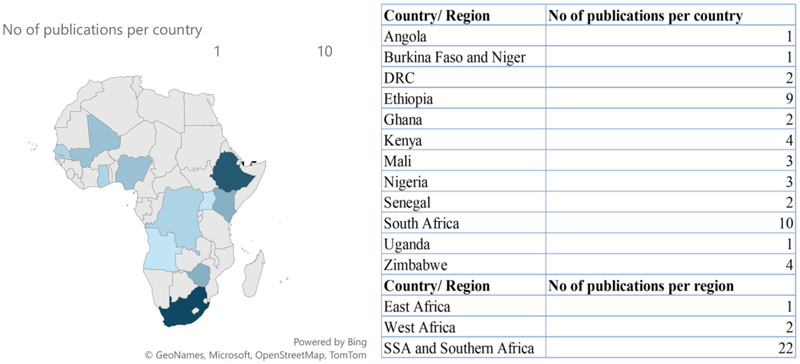
Visualization of the number of qualifying publications focusing on NUS from each country across SSA.

Publications from Scopus and Web of Science research databases totaled 1,545 (1,130 and 415) papers, and a total of 66 articles were included in this synthesis following a multi-stage comprehensive screening process on Covidence, as summarized in Figure 1. Following comments from an internal review, nine additional articles were added to support statements in the introduction and discussion sections. The literature included in the review synthesis totaled 75 publications. Research on the application of NUS and barriers hindering their use and adoption increased over the past decade. Approximately 56% of the articles included in the review were published between 2020 and 2023 (Figure 2), and the year with the most published literature on the topic was the year of the COVID-19 pandemic, 2020. South Africa had the most published literature on the subject matter, followed by Ethiopia, Kenya and other countries in SSA. Some publications, however, focused on the SSA region instead of individual countries (Figure 3). This section presents the results of the study qualitatively and quantitatively.

### 3.2 An analysis of the utilization and potential of NUS and NUS-inclusive local agri-food systems

#### 3.2.1 Food and nutrition security

Central to the reported importance of underutilized crops is their use and potential to be sustainable and intergenerational sources of food and affordable nutrition, especially in marginalized smallholder settings (Mabhaudhi et al., 2017a; Bilali, 2020; Hendre et al., 2019; Chivenge et al., 2015; Mabhaudhi et al., 2018b; Kodzwa et al., 2023; Chase et al., 2023; Mugiyo et al., 2021; van Zonneveld et al., 2021; Akinola et al., 2020; Mubaiwa et al., 2018; Agulanna, 2020; Cheng et al., 2017; Chemura et al., 2022; Paliwal et al., 2020; Catarino et al., 2021; Obidiegwu et al., 2020; Mbosso et al., 2020; Momanyi et al., 2020; Yemata, 2020; Muthoni and Nyamongo, 2010; Pushpalatha and Gangadharan, 2020; Diop et al., 2018; van Zonneveld et al., 2020; Morrow et al., 2023; Feyisa et al., 2022; Adejumobi et al., 2022). Because of this, NUS were acknowledged to directly contribute toward improving food security in 81.8% of the reviewed literature. This was supported by reports from 37.9% and 86.4% of the publications, which also acknowledged that these crops can indirectly enhance food security. NUS do so by enabling marginalized communities to engage in activities that improve their livelihoods and local agricultural systems respectively, thereby positively impacting their food security outcomes.

NUS crops are acknowledged to contribute toward the different pillars of food security, especially the availability of food, access and stability of the food systems as well as utilization, which, in enabling environments, supports positive nutritional outcomes (Hendre et al., 2019; Chivenge et al., 2015; Mabhaudhi et al., 2016; Kodzwa et al., 2023; Sidibé et al., 2020; Mugiyo et al., 2021; van Zonneveld et al., 2021; Agulanna, 2020; Cheng et al., 2017; Catarino et al., 2021; Mbosso et al., 2020; Yemata, 2020; Muthoni and Nyamongo, 2010; Pushpalatha and Gangadharan, 2020; Diop et al., 2018; Karunaratne et al., 2015; Mathews, 2010). It is also explicitly documented that in rural settings, NUS are known to improve socio-cultural outcomes (Mabhaudhi et al., 2017a, 2018b; Akinola et al., 2020; Tan et al., 2020), aid with fighting against hunger (Sirany et al., 2022; van Zonneveld et al., 2021; Mugiyo et al., 2021; Agulanna, 2020; Obidiegwu et al., 2020; Mbosso et al., 2020; Yemata, 2020; Shayanowako et al., 2021; Mondo et al., 2021; Paliwal et al., 2021) and supplement or have potential to aid climate change adaptation through improving production and productivity of agri-food systems, particularly under harsh conditions (86.4%) (e.g., Mabhaudhi et al., 2017a; Bilali, 2020; Chivenge et al., 2015; Mabhaudhi et al., 2016; Kodzwa et al., 2023; Nhamo et al., 2022; Chase et al., 2023; Zhang and Dannenberg, 2022; Paliwal et al., 2020).

NUS are reported to be natural insurance for food security in times of adversity. Authors like Yemata (2020) have gone as far as to report that communities that plant NUS species are generally food secure and have “*neve*r” known famine and that they are not exposed to extreme hunger or food insecurity even during harsh seasons in places like Ethiopia (Yemata, 2020). This claim was linked to underutilized crops’ characteristics and abilities to enhance food security and sufficiency through diversifying and intensifying local agri-food systems, particularly in water-scarce environments (Mabhaudhi et al., 2017a, 2018a, 2017b; Jiri et al., 2017; Muthoni and Nyamongo, 2010; van Zonneveld et al., 2020; Karunaratne et al., 2015; Woldeyohannes et al., 2020; Azam-Ali, 2007; Rhoné et al., 2020). In this way, NUS crops are strategic as it is well documented and acknowledged that reduced agricultural diversity has adverse consequences for food access, availability and security (Bilali, 2020; Jiri et al., 2017; Akinola et al., 2020; Paliwal et al., 2020; Mbosso et al., 2020; Woldeyohannes et al., 2020). Collectively, most NUS have been labeled as food security crops for rural farmers, particularly women, in settings where the main obstacle for agri-food systems and policymakers at different scales is to provide strategic solutions to the challenge of granting the growing population access to sustainable, healthy diets and ensuring food security, especially in rural areas (Mabhaudhi et al., 2017a; Bilali, 2020; Chivenge et al., 2015; Mabhaudhi et al., 2016; Kodzwa et al., 2023; Paliwal et al., 2020; Pushpalatha and Gangadharan, 2020; Diop et al., 2018).

#### 3.2.2 Food sovereignty

Progress toward designing and reinforcing quality food and nutrition-sensitive initiatives and approaches has been slow (Feleke and Tekalign, 2022; Sampson et al., 2021). Such approaches, for example, would be inclusive, and people-centered to inform equitable, sustainable agri-food systems transformation toward nutrition-sensitive systems that support food sovereignty and aim to reduce hunger and malnutrition and boost local economies, thus contributing to the five pillars of the SDG agenda, people, planet, prosperity, peace, and partnerships (Feleke and Tekalign, 2022; Sampson et al., 2021). Rural communities have relied on NUS to provide food systems stability, food sovereignty, dietary support, nutritional safety nets, food security and medicinal support to local communities for centuries during adverse climatic conditions (Agulanna, 2020; Obidiegwu et al., 2020).

Food sovereignty is essentially the right of people to define their own food systems (Sampson et al., 2021; Village and Sélingu, 2007). Central to the principle of food sovereignty is the people’s right to healthy, culturally accommodating, quality foods produced sustainably with ecologically sound values [62, Village and Sélingu, 2007]. The principle is inclusive and people-driven, with a mission to include local people as stakeholders in their agricultural food systems. Of the articles analyzed, 12.12% reported on the potential underutilized crops have to catalyze the efforts to reinforce food sovereignty and positive socio-cultural outcomes (Nhamo et al., 2022; World Health Organization, 2021; Azam-Ali, 2007; Handschuch and Wollni, 2016).

Underutilized crops not only have the potential to strengthen existing agri-food systems through agricultural crop diversification, but they can also strategically reinstate food sovereignty at local levels and promote dietary diversity, leading to better food and nutrition security outcomes (van Zonneveld et al., 2023; Mekonnen et al., 2022; van Zonneveld et al., 2020). NUS crops that remain in informal, indigenous smallholder farming systems are a product of intergenerational farmer selection and are supported by informal seed systems (Feleke and Tekalign, 2022; Mabhaudhi et al., 2017b). The farmers’ right to self-informed production and crop selection knowledge and preferences remain crucial to transform existing systems.

#### 3.2.3 Nutrition and health

**Figure 4:**
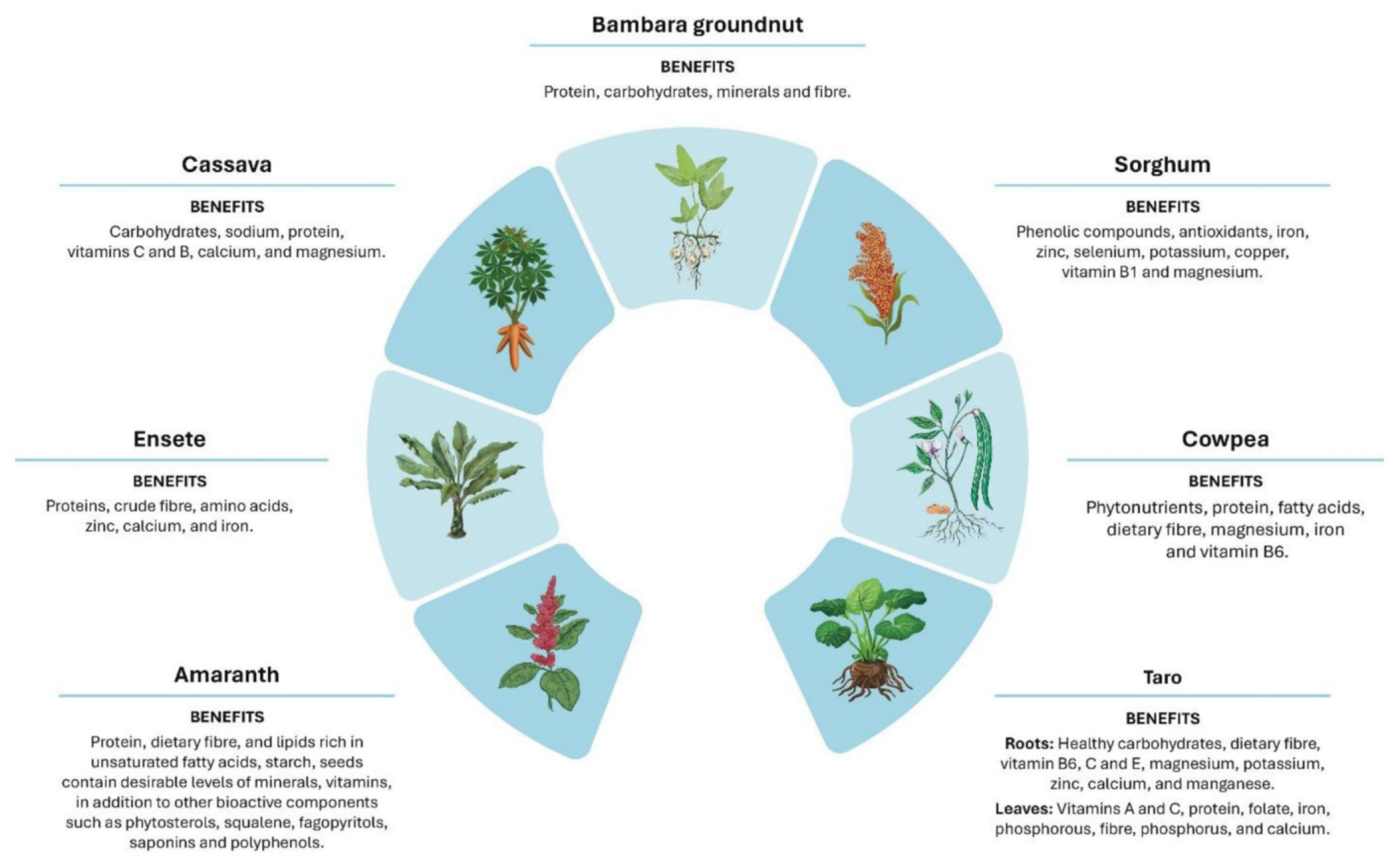
Nutritional benefits associated with several NUS presently prioritized in research.

Sub-Saharan Africa has among the world’s highest levels of hunger and micronutrient deficiencies, and this situation is worsening in the region faster than anywhere else in the world (Sirany et al., 2022; Hendre et al., 2019; Mekonnen et al., 2022; van Zonneveld et al., 2021; Rosenthal and Ort, 2012). Of the 36 countries with the highest scores of micronutrient deficiencies, 86% are in SSA (Hendre et al., 2019; Mekonnen et al., 2022). Further, non-communicable diseases (NCDs), malnutrition, stunting, and hidden hunger are all conditions that have been increasing in SSA following the shift to monoculture agri-food systems, and monotonous diets post the introduction of the Green Revolution and its framework (Mashamaite et al., 2022; Nkwonta et al., 2023; Zhang and Dannenberg, 2022). The Green Revolution and supporting frameworks and policies failed and continue to fail to account for food quality and nutrition security, leading to a decrease in agricultural and dietary diversity and neglected nutritional requirements of local populations in SSA (Hendre et al., 2019; Mabhaudhi et al., 2018b; Mugiyo et al., 2021; Mabhaudhi et al., 2017b; Agulanna, 2020; Hunter et al., 2019). The agri-food systems must be transformed and contextualized to cater to local needs (Mugiyo et al., 2021; Mabhaudhi et al., 2017b; Cheng et al., 2017). The re-introduction of indigenous crops to the broader population can be a strategic step to help address poverty and caloric and nutrition deficiencies (Mabhaudhi et al., 2017a; Abberton et al., 2022; Mabhaudhi et al., 2018b, 2016, 2017b; Jiri et al., 2017; Akinola et al., 2020; Agulanna, 2020; Cheng et al., 2017; Obidiegwu et al., 2020; Yemata, 2020; Muthoni and Nyamongo, 2010; Wang et al., 2014). SSA has a rich agrobiodiversity of NUS, which could be incorporated into transformed and contextualized agri-food systems that are food and nutrition security sensitive (Chivenge et al., 2015; Mabhaudhi et al., 2018b; van Zonneveld et al., 2023; Akinola et al., 2020; Agulanna, 2020; Muthoni and Nyamongo, 2010; Rhoné et al., 2020). The richness of NUS in nutrients, proteins, dietary fiber, healthy carbohydrates and proteins, vitamins, active compounds, antioxidants and minerals contributed and could contribute toward improving the health statuses of people at different scales (Figure 4) (Hendre et al., 2019; Chivenge et al., 2015; Kodzwa et al., 2023; Agulanna, 2020; Obidiegwu et al., 2020). Underutilized crops have the potential to improve access and availability of diverse, nutritious and healthy foods that can improve nutritional and health outcomes and quality of life in populations at different life stages and regional scales (Hendre et al., 2019; Mabhaudhi et al., 2018b; Obidiegwu et al., 2020).

### 3.3 NUS and their prioritization in research

Underutilized crops can be considered strategic foods to solve numerous challenges in SSA under current and future conditions (Popoola et al., 2022; Abberton et al., 2022; Hendre et al., 2019; Sidibé et al., 2020; Akinola et al., 2020). In the literature included in this synthesis, several NUS were reported to be more important and strategic because of their characteristics and potential to contribute toward solving the region’s prevailing problems. This section reports on the “strategic” NUS crops currently prioritized in research for their specific characteristics and potential to improve adaptive capacity and sustainability under climate change and food and nutrition security outcomes. However, processing constraints bottleneck their sustainable deployment in mitigating food and nutrition insecurity (Mubaiwa et al., 2018; Diop et al., 2018). Existing literature expounds that post-harvest handling and processing techniques of NUS species are limited to traditional methods and lack innovation to improve their shelf life and taste without interfering with the nutritional value of the end products (Mubaiwa et al., 2018; Mbosso et al., 2020; Diop et al., 2018).

#### 3.3.1 Priority underutilized crops

**Figure 5:**
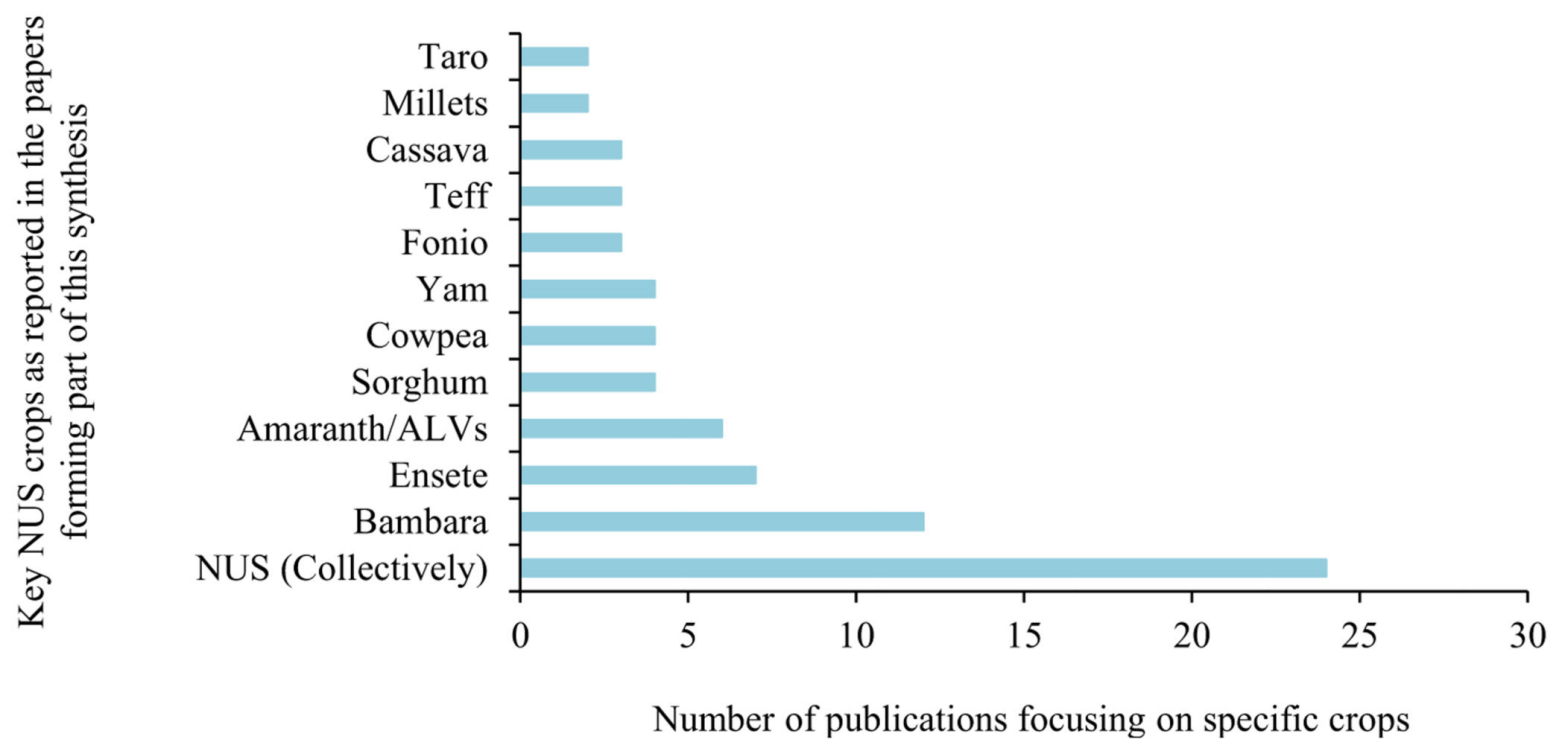
Top 11 most researched NUS based on the reviewed literature published between 2000 and 2023.

**Figure 6:**
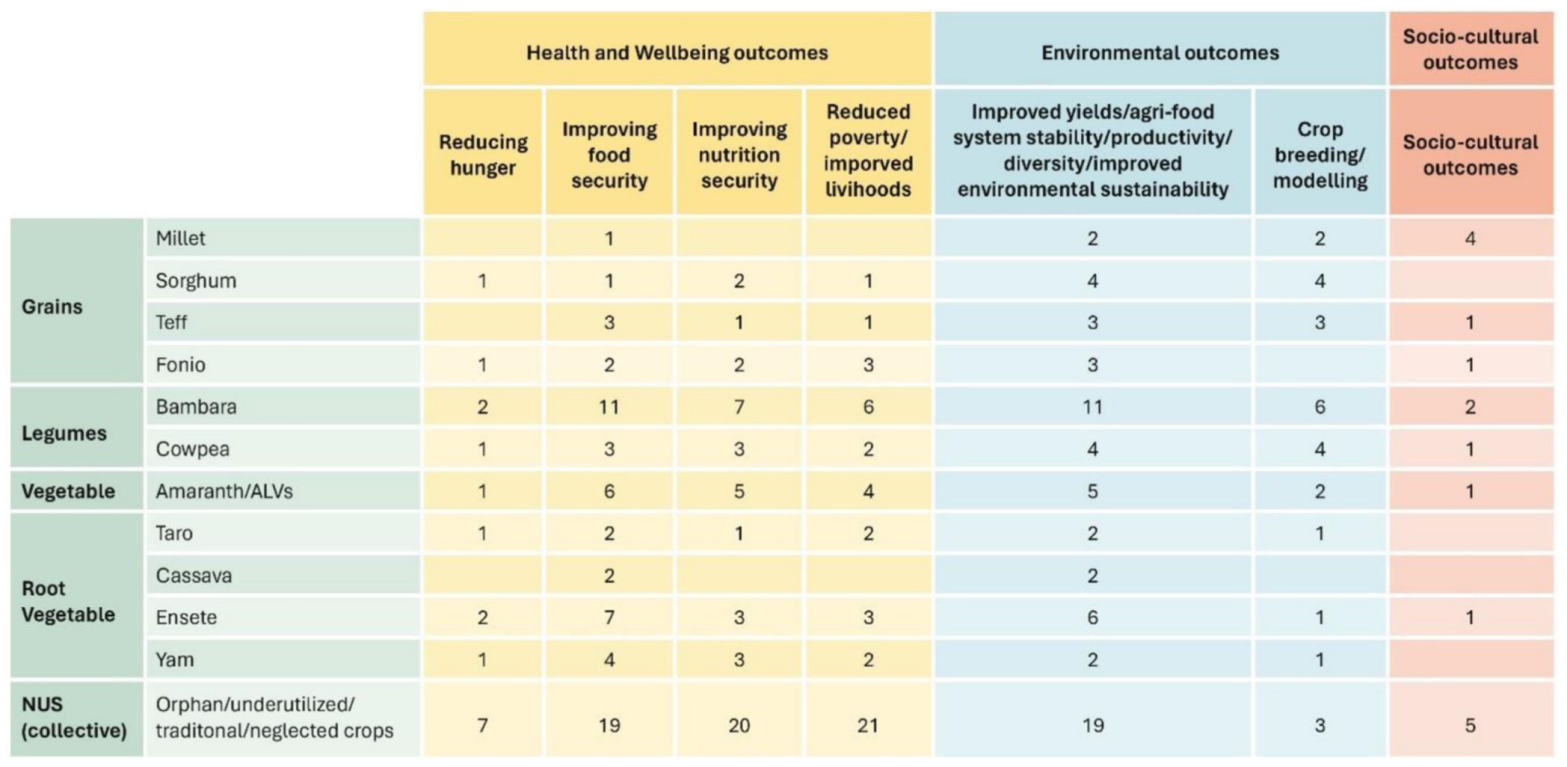
Evidence heat map showing the prevalence of outcomes within included publications focusing on NUS in SSA. The health and wellbeing and environmental outcomes present a collection of specific sub-outcome proportions.

**Figure 7:**
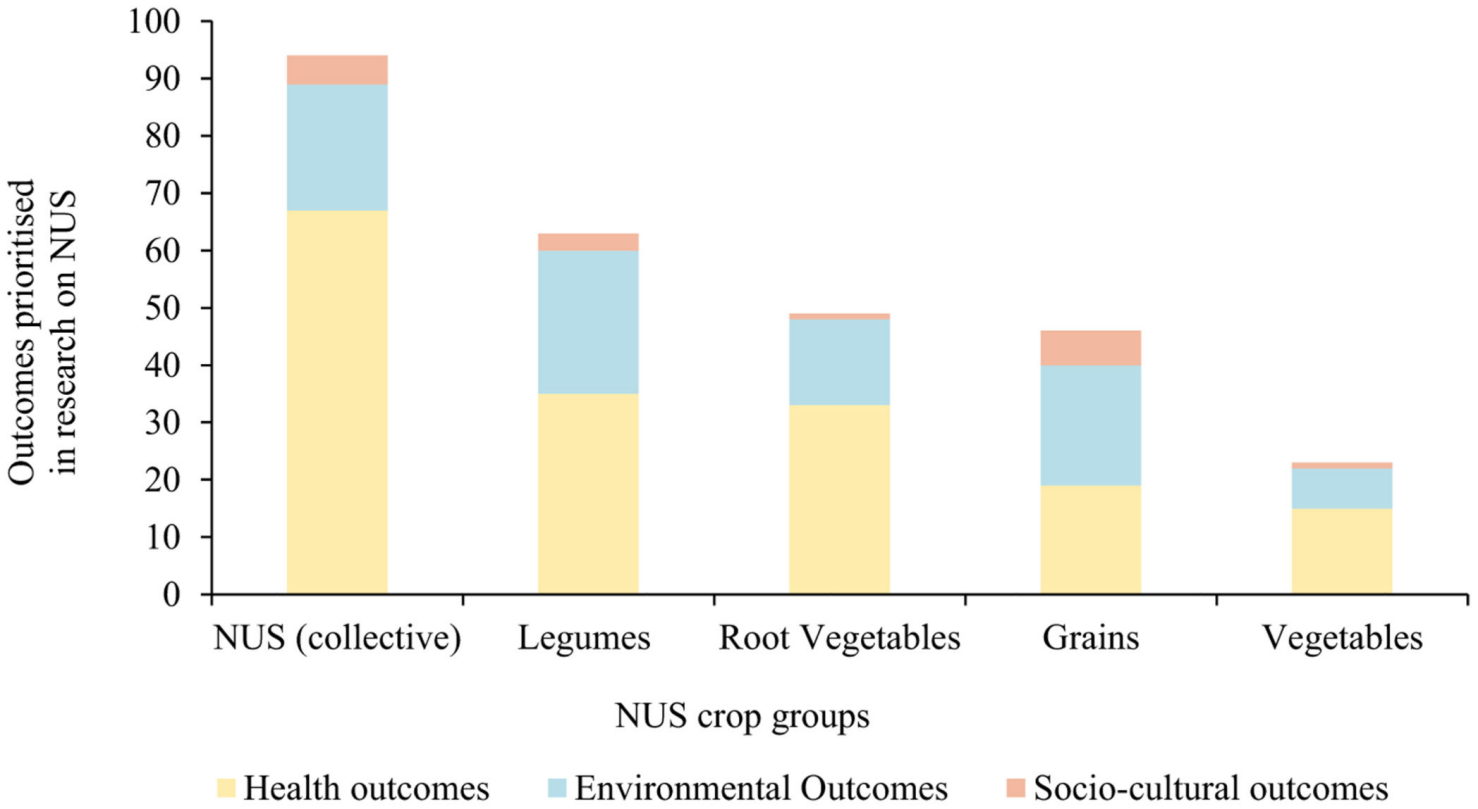
Frequency of each reported outcome per NUS group.

The studies reviewed highlight the importance and potential of bambara groundnut (*Vigna Subterrane*) as a strategic NUS crop in 18.18% of the reviewed publications. Significant evidence also highlighted the importance of ensete (*Ensete ventricosum*), in 10.66% of the literature reviewed. In comparison, modest evidence on amaranthus and other African leafy vegetables (9.09%), cowpea (*Vigna unguiculata*), sorghum and yam (*Dioscorea L)* (6.06% of the publications each) highlighted the potential and benefits of including these crops in local agricultural systems as a climate change, food and nutrition security, and local livelihood adaptation strategy. cassava *(Manihot esculenta Crantz*), fonio *(Digitaria exilis*) and teff (*Eragrostis tef)* (4.54% of publications each) and millet (*Pennisetum glaucum*) and taro (*Colocasia esculenta*) were also included among the underutilized crops with the potential to aid in addressing existing challenges in the region with limited evidence base (3.03% of included publications) supporting them as strategic crops (Figure 5). Twenty-four (Mekonnen et al., 2022) publications included in this synthesis grouped these NUS crops and reported on them collectively. Collectively, these crops were identified as having the potential to sustainably contribute toward enhancing agri-food systems productivity and stability that way, improving food and nutrition security and socio-economic, socio-cultural and environmental outcomes in SSA (Figures 6, 7) (Popoola et al., 2022; Abberton et al., 2022; Mudau et al., 2022; Chivenge et al., 2015; Mabhaudhi et al., 2016; Sidibé et al., 2020; van Zonneveld et al., 2023; Chase et al., 2023; Mugiyo et al., 2021; Zhang and Dannenberg, 2022; Agulanna, 2020; Koch et al., 2021; Paliwal et al., 2020; Catarino et al., 2021; Mbosso et al., 2020; Muthoni and Nyamongo, 2010; Morrow et al., 2023; Karunaratne et al., 2015; Paliwal et al., 2021; Azam-Ali, 2007; Handschuch and Wollni, 2016; Alemayehu et al., 2015; Majola et al., 2021; Mugambiwa, 2018).

NUS crops have also been reported to have numerous cultural and medicinal properties. They can contribute toward the stability and diversity of agri-food systems at different scales, re-informing food sovereignty, environmental sustainability and ecosystem services through improving agro-biodiversity (van Zonneveld et al., 2020; Karunaratne et al., 2015; Azam-Ali, 2007; Handschuch and Wollni, 2016; Mugambiwa, 2018). Through their different properties, underutilized crops are strategic to catalyze the region’s progress toward achieving SDGs, particularly SDGs 1, 2,3 and 15 and contributing to the five pillars of the SDG 2030 agenda, through informing and intensifying:

##### 3.3.1.1 Building resilient agri-food systems

A selected few major crops dominate the current agri-food systems, with three specific major crops having been reported to utilize up to 40% of all the arable land globally post-2011 (Mekonnen et al., 2022). Increasing agricultural biodiversity can minimize the risk and vulnerability of local agri-food systems and livelihoods, reinforce production stability and resilience, and minimize risks to food and nutrition security in the region (van Zonneveld et al., 2020; Pironon et al., 2019; Alemayehu et al., 2015). Research has reported on the potential NUS for the past years, such as those in Figure 7, have to contribute toward agricultural improvements and mitigation and adaptation measures in the agri-food systems and the resilience of these systems given climate change and environmental changes (Mabhaudhi et al., 2017a; van Zonneveld et al., 2021; Akinola et al., 2020; Paliwal et al., 2020; Catarino et al., 2021; Mbosso et al., 2020; Feyisa et al., 2022). Their potential expands from reports on their adaptability to changes and adversity and suitability to a wide range of environments, easy maintenance, and fewer input requirements (Mabhaudhi et al., 2017a; Popoola et al., 2022; Abberton et al., 2022; Mabhaudhi et al., 2016; Sidibé et al., 2020; Nhamo et al., 2022; Mabhaudhi et al., 2017b; Mbosso et al., 2020; Morrow et al., 2023; Feyisa et al., 2022; Paliwal et al., 2021; Woldeyohannes et al., 2020; Majola et al., 2021). These crops are also favorable due to their tolerance to biotic and abiotic stressors and ability to withstand harsh conditions and intensify agri-food systems, especially in rural areas (Bilali, 2020; Mabhaudhi et al., 2018a; Akinola et al., 2020; Paliwal et al., 2020; Catarino et al., 2021; Mugambiwa, 2018). Some of these crops, for example, Bambara groundnut, can further contribute toward nitrogen fixation and encourage increased yields in farming systems that would otherwise struggle (Sidibé et al., 2020; Jiri et al., 2017).

Underutilized crops can also contribute toward improvements of agri-food systems through genetic advancement of some major crops, helping them become more adaptable and resilient to future and present climatic and environmental changes (Abberton et al., 2022; Mudau et al., 2022; Hendre et al., 2019; Cheng et al., 2017; Paliwal et al., 2020; Catarino et al., 2021; Obidiegwu et al., 2020; Muthoni and Nyamongo, 2010; Pushpalatha and Gangadharan, 2020; Woldeyohannes et al., 2020). Because of this, some publications highlight the importance of conserving some NUS, particularly those mentioned above, for example, Bambara groundnut, which was said to be at risk of abandonment and genetic erosion as a result of their marginalization and elimination from agri-food systems despite their potential because of their poorly defined value-chains and informal seed systems (Chivenge et al., 2015; Paliwal et al., 2020; Catarino et al., 2021; Obidiegwu et al., 2020; Mbosso et al., 2020). However, like most NUS, these crops have been neglected in developmental agendas, food systems policies, and seed systems formalization efforts (Sidibé et al., 2020; Cheng et al., 2017).

One of the main strategies to improve existing agri-food systems and agricultural adaptation and resilience to shocks is perceived to be the development of more resilient crop varieties of some major crops using genetic traits of NUS as well as diversification of agrobiodiversity through the inclusion and promotion of NUS crops (Cheng et al., 2017; Paliwal et al., 2020; Catarino et al., 2021; Obidiegwu et al., 2020; Woldeyohannes et al., 2020). However, there are limited efforts to support this. Genomics breeding, molecular breeding, genetic engineering and using traits of NUS to improve some major crops genetically and promote phenotypic diversification for improved food-nutrition security have potential as strategies for agricultural climate change adaptation (Abberton et al., 2022; Hendre et al., 2019; Sidibé et al., 2020; Cheng et al., 2017; Paliwal et al., 2020; Catarino et al., 2021; Woldeyohannes et al., 2020; Paff and Asseng, 2019; Singh et al., 2014; Faye et al., 2019). NUS have phenotypic diversity to support agricultural production and improve agricultural diversification under climate change conditions (Chivenge et al., 2015; Akinola et al., 2020; Paliwal et al., 2020; Catarino et al., 2021; Obidiegwu et al., 2020; Woldeyohannes et al., 2020).

##### 3.3.1.2 Socio-economic benefits

Sub-Saharan Africa’s share in the global food system, agricultural exports, and local food sovereignty drastically decreased following the Green Revolution and supporting systems (Mabhaudhi et al., 2017a; Hendre et al., 2019; Mabhaudhi et al., 2018b; van Zonneveld et al., 2021; Akinola et al., 2020; Azam-Ali, 2007; Alemayehu et al., 2015). The Green Revolution improved productivity and production of specific crops, largely “cash crops”, and seemed to have contributed toward progress made to achieve food security and suppressing global hunger levels between 1990 and 2014 (Food Agriculture Organization, 2015). However, the Green Revolution systems largely failed in SSA due to their oversight to address context-specific requirements of a sustainable and holistic food system, local socio-economics value chains, side-lining the role of local climates in the sustainability of agri-food systems, existing ways of life, and other socio-economic and socio-cultural requirements (Hendre et al., 2019; Mabhaudhi et al., 2018b; Agulanna, 2020). Existing local and traditional agricultural value chains were primarily displaced by the efforts and framework of the Green Revolution, even though the objective of improving productivity and intensification of food production through streamlining the food system and mainstreaming higher-yielding crops was achieved (Hendre et al., 2019; Mabhaudhi et al., 2018b, 2016, 2017b; Hunter et al., 2019).

Post the Green Revolution, reduced agricultural diversity and monocultural cropping systems that support input-driven major crops that are not as locally suited and adaptable as landraces and previously grown varieties, are the single most prominent challenges threatening socio-economic statuses, livelihoods, food security, human health and nutrition sectors in SSA (916; Akinola et al., 2020; Veldsman et al., 2023; Zhang and Dannenberg, 2022; Hunter et al., 2019). The system exacerbated inequality, poverty, food and nutrition insecurity and environmental challenges. However, climate change impacts will be more severe in the regions in developing nations where poverty, malnutrition and food insecurity are already problematic and adaptive capacity is low, with limited resources to cope with external threats and shocks (Rosenthal and Ort, 2012; Alemayehu et al., 2015).

Most NUS are healthier alternatives to staple crops, have multiple socio-economic and socio-cultural benefits, and can be produced in an ecologically friendly way (Feleke and Tekalign, 2022; van Zonneveld et al., 2023; Mashamaite et al., 2022; Nkwonta et al., 2023; Morrow et al., 2023; Adejumobi et al., 2022). For generations, these species have been reported to be “natural insurance” for hunger and food security (Hendre et al., 2019; Mugiyo et al., 2021; Akinola et al., 2020; Agulanna, 2020; Obidiegwu et al., 2020; Mbosso et al., 2020; Yemata, 2020). Underutilized crops are also comprehensively reported to be sources or potential sources of income generation, poverty eradication and natural insurance for local livelihoods, which means their incorporation can assist the region’s efforts to achieve SDG 1 of zero poverty SDGs 2,3, and 15 (Mabhaudhi et al., 2017a; Bilali, 2020; Mabhaudhi et al., 2018b, 2016; Akinola et al., 2020; Agulanna, 2020; Catarino et al., 2021; Mbosso et al., 2020; Momanyi et al., 2020; Karunaratne et al., 2015). People who currently benefit from using NUS for socio-economic reasons are primarily women from poor marginal areas. They are considered significant in the current value chain of NUS and are labeled as guardians and conservators of NUS and their seed systems (Bilali, 2020; Mabhaudhi et al., 2016; Sidibé et al., 2020; Agulanna, 2020). This highlights gaps where government needs to come in to assist local efforts that ensure conservation and utilization of NUS crops. Table 2 summarizes some of the reported socio-economic values of NUS.

### 3.4 Research outcomes and gaps in NUS prioritization

Most of the publications reviewed advocate for NUS as a solution to the region’s food and nutrition insecurity challenges and highlight the characteristics that make NUS important to be featured as a component of informing agri-food systems transformation. Mabhaudhi et al. (2017b) highlighted existing gaps and provided recommendations on how to address existing gaps and realignment of policy focus to promote NUS prioritization and application, however, efforts toward this remain stagnant (Abberton et al., 2022; Mudau et al., 2022). Our review further advanced these gaps by identifying that health and wellbeing, and environmental outcomes were prioritized in research involving NUS, while sociocultural outcomes, although acknowledged, were not prioritized. The studies that collectively reported on NUS focused primarily on health and wellbeing outcomes, poverty reduction and strengthening livelihoods, nutrition and food security and environmental outcomes, respectively (Figures 6, 7). Root vegetables and legumes were specifically linked to research prioritizing health outcomes, while grains were largely linked to environmental and socio-cultural outcomes. This reflects a focus on promoting NUS based on their environmental and nutritional benefits and less on their socio-cultural importance. A gap, therefore, exists regarding addressing social stigmas associated with NUS to enable the potential of these crops to be realized (Mabhaudhi et al., 2017b). Policy revisions need to be undertaken to widen the scope of NUS prioritization in research and enable their acceptance and application at different scales.

### 3.5 Barriers to the adoption and utilization of NUS

NUS are comprehensively reported to have the potential to address several socio-economic and cultural challenges (e.g., Table 2). The potential of NUS stems from their characteristics which presents them strategically as a constituent solution to catalyze efforts to address food and nutrition challenges and contribute toward resilience of local agri-food systems and livelihoods (Sidibé et al., 2020; Nhamo et al., 2022; Mekonnen et al., 2022; Mbosso et al., 2020; Shayanowako et al., 2021; Majola et al., 2021; Paff and Asseng, 2019; Faye et al., 2019). However, several barriers currently exist and prevent NUS from transitioning from being underutilized crops toward becoming more mainstream. These systematic and entrenched barriers make translating the goodwill on NUS into actionable outcomes difficult. On the other hand, these barriers are enabled by the marginalization of these crops in research, production and developmental initiatives. Existing barriers are further enabled by limited data availability on NUS production and processing techniques, misaligned research and policy priorities, their neglect of genetic improvement efforts and lack of efforts promoting them as a potential key component to agri-food systems transformation, particularly under climate change (Momanyi et al., 2020). The main barriers are highlighted below:

### 3.6 Insufficient agricultural innovation

The correlation between NUS and climate change mitigation and adaptation within the agricultural and food systems to support food security and stability, nutrition security and health exists (Mabhaudhi et al., 2016; Sidibé et al., 2020; van Zonneveld et al., 2021; Jiri et al., 2017; Paliwal et al., 2020; Catarino et al., 2021; Woldeyohannes et al., 2020; Mugambiwa, 2018). However, research and data on indigenous and genetic characteristics of NUS is still scattered and limited (Sirany et al., 2022; Abberton et al., 2022; Mabhaudhi et al., 2016; Mbosso et al., 2020). Management strategies, and production information of these crops often be passed down through local generations in vernacular systems rather than published and conserved systems (Chase et al., 2023; Alemayehu et al., 2015). This translates to a lack of knowledge of these crops, misaligned research, breeding and seed testing priorities, the marginalization of NUS crop species in research and developmental agendas and missed opportunities to utilize these crops to solve regional challenges (Chivenge et al., 2015; Mekonnen et al., 2022).

Further, informal, variable and inequitable seed systems, alongside the capitalized seed bills and frameworks which criminalized seed exchange and free will when it comes to seed selection of some “unregistered crop varieties”, which are primarily underutilized crops are other major production and adoption bottlenecks for these species (Chivenge et al., 2015; Sidibé et al., 2020; Mondo et al., 2021). Additionally, reports of NUS being less yielding and less commercially viable due to limited uses and ill-defined and underdeveloped value chains and seed systems are obstacles to adopting these crops (Sirany et al., 2022; Popoola et al., 2022; Bilali, 2020; Sidibé et al., 2020; Mekonnen et al., 2022).

The ideological, structural and institutional barriers put in place to support the production of major crops post the Green Revolution discourage local communities from incorporating and embracing NUS crops in their agri-food systems. Transforming local agri-food systems requires ethical consideration of traditional knowledge systems, cultural needs, climate and general inclusivity. Therefore, these barriers need to be overcome. Traditional and scientific knowledge systems and innovations on NUS agronomic requirements and processing must align to promote NUS. Additionally, well-developed seed systems, markets and efforts to destigmatize these crops, enabling their adoption and creating competitiveness for NUS cultivators in the agri-food systems value chains at different scales, are necessary (Chivenge et al., 2015; Sidibé et al., 2020; van Zonneveld et al., 2021). These efforts, however, are currently limited.

### 3.7 Gendered restrictions

Women, especially in rural areas, are reported to be the primary guardians and stewards of NUS species (Catarino et al., 2021). They, however, lack support and inclusion in developmental agendas to promote these crops, which are still, in some instances, gendered and labeled as “animal feed and poor farmers’ crops and food” (Sirany et al., 2022, p. 3). This continues to hinder the potential of NUS to contribute to reinstating food sovereignty, food and nutrition security and socio-economic improvements, especially in rural settings (Sirany et al., 2022; Bilali, 2020; Catarino et al., 2021).

To date, genetic resources improvement, seed systems, and the broader value chain of NUS and the currently limited NUS products are marginal and managed, sustained and fuelled within rural communities by primarily women (Chivenge et al., 2015; Catarino et al., 2021; Mondo et al., 2021; Alemayehu et al., 2015). This, because of gender dynamics and other socio-economic and cultural barriers, leads to an ongoing cycle of informalized seed systems and NUS crop value chains. This situation would be different with correct institutional support, properly aligned research priorities, improved genetic resources and ensuring that seed systems of these crops are managed and conserved through formal seed production institutions (Abberton et al., 2022; Bilali, 2020; Paliwal et al., 2021; Mondo et al., 2021).

### 3.8 Biophysical barriers and natural attributes

Some NUS, because of their biophysical characteristics, have been widely reported to lack preferred consumer attributes. These attributes, for example, are reported to be high-yielding, easy to process, have shorter cooking time, have familiar/favorable taste, and require less water and minimal energy when processing (Sirany et al., 2022; Mekonnen et al., 2022; Mubaiwa et al., 2018). Neglect of these crops has translated to them not having improved high-yielding varieties and they lack modernized processing techniques, inputs and necessary promotion and have limited market access (Chivenge et al., 2015; Mekonnen et al., 2022; Shayanowako et al., 2021; Mondo et al., 2021). Underutilized crops, in some cases, as cultural crops lack, but require innovations of culturally acceptable, feasible and nutrient-sensitive processing techniques to improve their adoption within different settings (Mubaiwa et al., 2018; Mondo et al., 2021).

## 4 Discussion

Research on NUS has increased steadily over the past 23 years and more exponentially since 2015, highlighting a linkage between the increasing interest in NUS and the United Nations’ Sustainable Development Goals (SDG) agenda. This review outlines various perspectives on the utilization and potential of NUS to achieve sustainable, equitable, inclusive, resilient and diversified agri-food systems and reinforce food sovereignty within safe planetary boundaries under climate change in SSA. Publications on the use and potential of NUS are from multiple perspectives, ranging from sustainability and environmental research and climate change to food science and nutrition research. This highlights NUS’ transformative and integrative potential to contribute to a broader sustainability agenda that cuts across systems and scales, particularly after the COVID-19 pandemic.

This review further highlights significant barriers to NUS adoption, often contributing toward the status of these species remaining underutilized at different levels. The review highlighted the lack of integrated knowledge and seed systems, ill-defined value chains at different scales, gendered restrictions, and natural or biophysical challenges as the main barriers to NUS production. Other challenges included socio-cultural and structural ideologies. All of these are major bottlenecks hindering the adoption of NUS and better positioning of these crops in developmental agendas and adoption for utilization and are an outcome of their lack of inclusion and misaligned priorities between research, genetic improvements and policies (Table 3) (Mabhaudhi et al., 2017a; Sirany et al., 2022; Chivenge et al., 2015; Mabhaudhi et al., 2018b; Mekonnen et al., 2022; Akinola et al., 2020).

Food security is defined as a situation where “*all people, at all times, have physical, social, and economic access to sufficient, safe, and nutritious food that meets their food preferences and dietary needs for an active and healthy life*” (Chivenge et al., 2015, p. 5686). Climate change reduced agricultural diversity, and biodiversity loss negatively affect food and nutrition security outcomes because access constraints to diverse food options and choices vector food-nutrition and health-related challenges.

Further, reports highlight that climate change has evolved into a climate crisis with devastating effects on food production and production security, supply, availability, accessibility, and human livelihoods, especially in SSA (Jiri et al., 2017; Paliwal et al., 2020; Mbosso et al., 2020; Woldeyohannes et al., 2020; Pironon et al., 2019). Most of the reviewed literature highlights and advocates for NUS as a solution to these challenges, especially in rural areas most vulnerable to climate crises and resulting production challenges, poverty, and food and nutrition insecurity challenges (Mabhaudhi et al., 2018b; Nhamo et al., 2022; Akinola et al., 2020; Zhang and Dannenberg, 2022; Mathews, 2010; Azam-Ali, 2007).

### 4.1 Policy and practice: challenges and a need for transformation

Global conversations and action plans on climate change adaptation options for developing countries, focusing on agri-food systems, need to prioritize benefits for human nutrition to bridge existing food and nutritional gaps (Hendre et al., 2019). Future strategies, policies and action plans must acknowledge the importance of intersectoral collaborations for environment, food production and nutrition strategies as they bring co-benefits. Policy interventions also need to support the formalization of systems to improve the knowledge base and availability of priority NUS. For example, effective seed systems management is crucial for agricultural improvements and climate change adaptation. Policies should also focus on funding and support from country-specific governing institutions, as well as ethical communication and promotion to destigmatize NUS. Previous strategies have proven short-sighted and have yet to be holistic, sustainable, and inclusive. They are limited in their account of environmental and cultural sensitivity and local populations’ nutritional and health aspects and needs.

Most barriers to NUS adoption and improved utilization can be addressed through realigning research and policy priorities. In SSA, for example, as a result of misaligned priorities and the lack of integration in policy, research and adaptation strategies, the number of malnourished people has been increasing post-2014, and the hidden hunger index is worsening in the region faster than anywhere else in the world (Sirany et al., 2022; Mekonnen et al., 2022). The re-introduction and inclusion of NUS crops in local agri-food and seed systems to support sustainable and resilient production and productivity and improved agricultural biodiversity of farming systems, particularly marginalized farming systems within safe planetary boundaries, can also aid in addressing these barriers (Chase et al., 2023; Alemayehu et al., 2015; Pironon et al., 2019). Table 3 summarizes drivers of adoption and reported barriers to NUS adoption.

The main challenges underlying the shortcomings and failure of the current agri-food systems, food and nutrition insecurity and health challenges in the region are familiar within the policy space. However, the efforts to address them have stagnated. Existing key regional policies and strategies put in place, such as the SADC Regional Indicative Strategic Plan (RISDP) of 2003 (www.sadc.int, n.d.), African Union Agenda 2063 (*Agenda 2063: The Africa We Want*, African Union, 2013; African Union Development Agency, n.d.) Comprehensive Africa Agricultural Development Programme (CAADP) (African Union Development Agency, n.d), Regional Agricultural Policy (RAP) (www.nepad.org, n.d.a,n), African Union Malabo Declaration on Accelerated Agricultural

Growth acknowledge all challenges underlying food and nutrition insecurity in SSA and put agricultural development at the center of addressing existing challenges (www.nepad.org, n.d.a,n). Supporting these are country-specific strategies for climate change adaptation and national development and disaster management plans, all calling for actions toward ending food and nutrition insecurity through the transformation of the current, outdated agri-food systems, which lack resilience and translated to one of the underlying contributors to food and nutrition insecurity in the region (Sirany et al., 2022; Mekonnen et al., 2022; Paliwal et al., 2021).

However, there needs to be more explicit mention of a need for food and nutrition-sensitive innovations and food sovereignty within the existing agricultural developmental policies, plans and frameworks. While food and nutrition insecurity are mentioned in the policy documents as two challenges that need to be addressed, clear revisions to be made in the agri-food systems in contextualized settings in the region are lacking. The implementations of interventions designed to address challenges relating to food and nutrition insecurity also come short due to a need for more contextualization in these interventions to address area-specific challenges. For example, the progress made by the Malabo Declaration for Agriculture transformation in Africa is slow and not sufficient in vital components of the declaration: Ending hunger by 2025, Agricultural investments and Improving resilience to Climate change and variability in many countries in the region (www.nepad.org, n.d.a,n).

While the need for financial support and services is well documented, an urgent need within existing policies and plans would be strengthening non-financial assets and services to avoid the ill-implementation of interventions at different scales. In a region like SSA, where financial poverty prevails, agricultural-developmental policies should integrate aspects of financial policies (Mabhaudhi et al., 2017b; Nkwonta et al., 2023). This should underly an environment that aims to bridge financial gaps within the agricultural value chains by being comprehensive, equitable and inclusive enough to accommodate the poor farmers and current NUS conservators and grant them access to financial services to support their efforts.

Underutilized crops have been safety nets for rural communities for nutrition, food security, and food sovereignty for centuries under diversified, adverse climatic conditions (Mashamaite et al., 2022; Paliwal et al., 2020). However, the cultivation of NUS has been declining over the years as more farmers abandon these species for highly promoted and policy-supported commercial crop species and changing eating habits (Mabhaudhi et al., 2017a; Sidibé et al., 2020). One of the main focuses of agri-food systems policies should be to reinforce food stability and ensure food availability, access, and affordability of healthy, nutritious food, as well as improve the food sovereignty of locals at different scales. Underutilized crops offer an opportunity for this to be realized.

The potential for NUS to address existing challenges is widely researched and documented. However, their inclusion is hindered by a lack of structures and policies to support their adoption and implementation of processes that could enable their inclusion (Mabhaudhi et al., 2017b; Nkwonta et al., 2023; Veldsman et al., 2023; Tan et al., 2020). Policy biases, for example, within the current agricultural and seed systems, expose the lack of alignment to address existing and projected challenges relating to food and nutrition insecurity and inequality and inequity in policy across SSA. In this way, current policies undermine local food sovereignty and an opportunity to bridge existing food and nutrition-related challenges (Mabhaudhi et al., 2017b; Nkwonta et al., 2023). Existing policies have been supporting smallholder agriculture and farmers through, for example, subsidizing inputs. However, these efforts continue to support and reinforce the acceptability and adoption of outdated and failing agri-food systems instead of accommodating and supporting transformation toward feasible, context-specific, inclusive, resilient and sustainable systems. This is made visible by the existing gaps and biases in policy.

**Figure 8:**
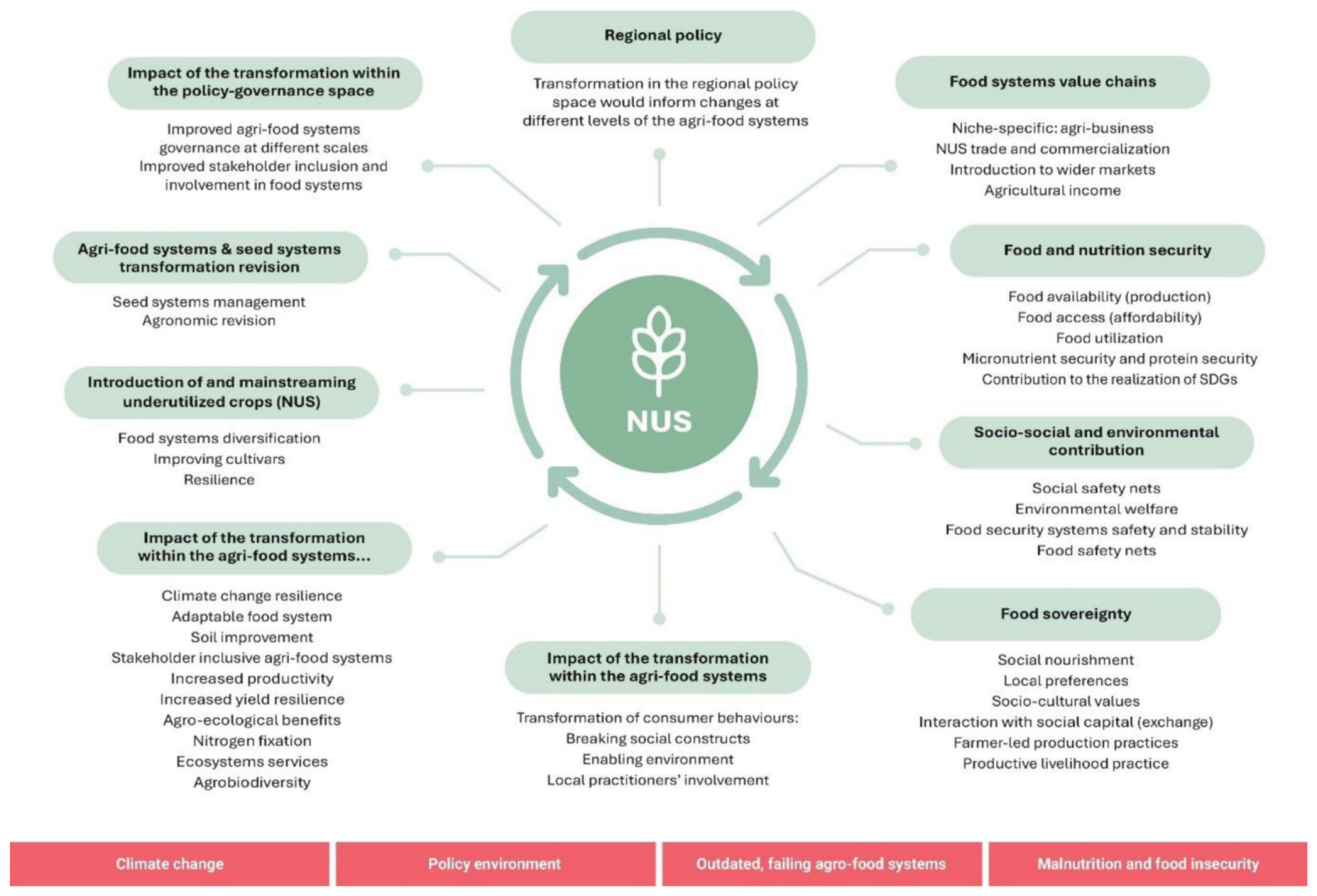
A conceptual framework for NUS-sensitive policy transformation to enable agri-food systems transformation to improve the state of malnutrition and food insecurity in SSA within safe planetary boundaries. The framework also highlights a gap where different stakeholders at different governance levels including NGO, development partners and similar can come in as role players in NUS-focused agri-food systems transformation. Framework conceptualized by the authors.

A need for policy revisions, interdisciplinarity, cross-sectoral collaboration, and coherence in the agri-food-nutrition-health systems is urgent, and the window for action is narrowing. The inclusion of NUS as a sustainable and resilient option for reinstating food sovereignty, transforming existing agri-food systems, and mitigating the impacts of climate change in the food and nutrition sectors needs to be prioritized as a strategy in the policy space. However, it should be an important and strategic component for transforming food systems from monopolized toward inclusive space and reinforcing food sovereignty for improved food and nutrition security, particularly within the marginalized communities in SSA (Figure 8).

### 4.2 Theoretical framework: enabling agri-food systems transformation by incorporating NUS

Current agri-food systems have contributed to multiple environmental and socio-economic challenges in SSA. Transforming these agri-food systems through incorporating alternative and contextualized systems that are sustainable, inclusive and serve within safe planetary boundaries will have multiple benefits for the environment and different aspects of human life, particularly food and nutrition security. There is growing evidence that NUS have substantial potential to solve existing socio-economic challenges in the region and contribute to climate change mitigation, adaptation and resilience of the region’s agri-food systems. However, there is a gap in actualizing this potential and successfully mainstreaming NUS into agrifood systems.

**Figure 9:**
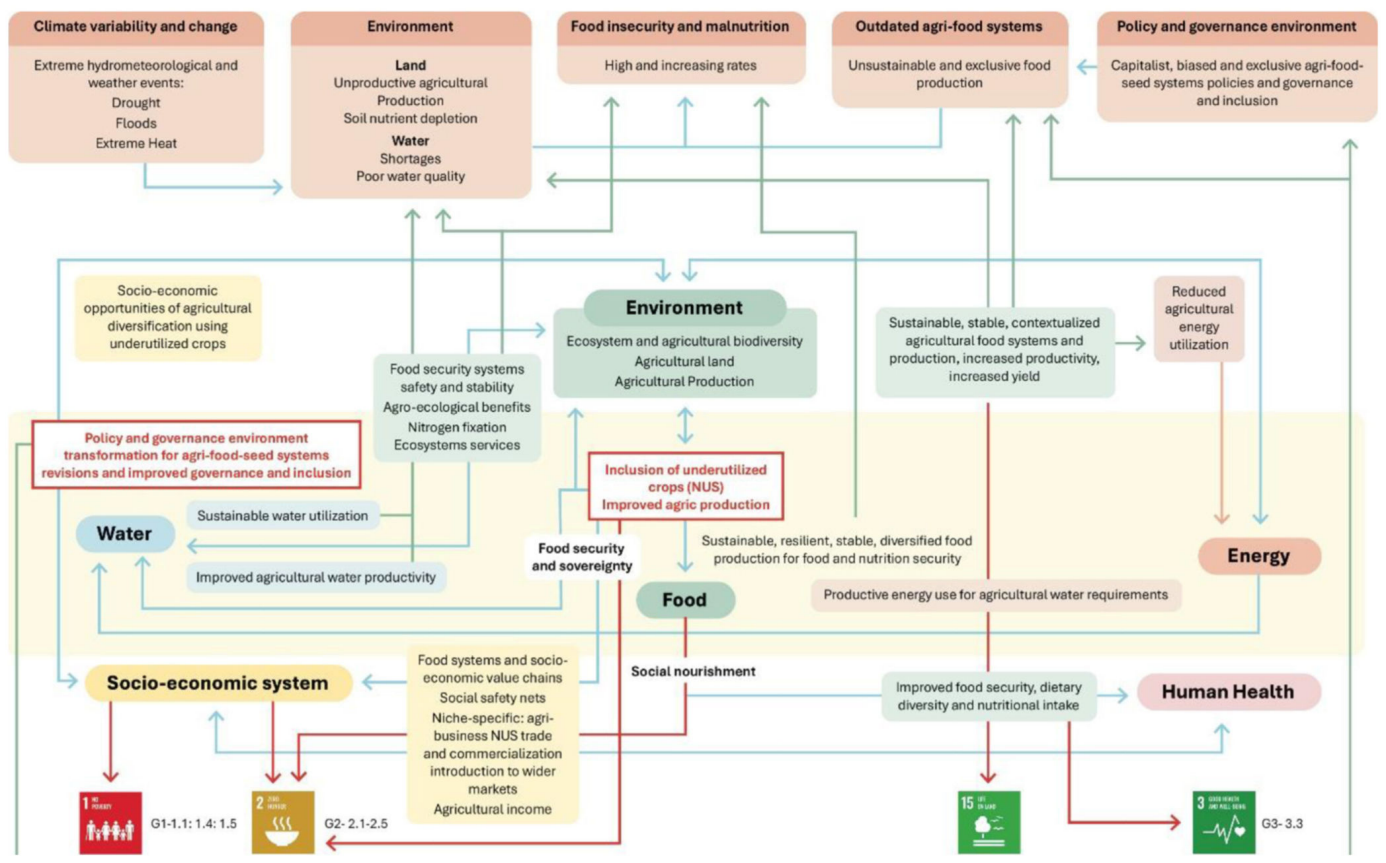
Synthesis of linkages between existing challenges in SSA and the potential of NUS to address these challenges, which fall under SDG priority areas, particularly SDGS 1, 2, 3, and 15. Source: Framework conceptualized by the authors based on review findings.

The framework presented in Figure 9 conceptualizes regional challenges, a need for transformation and the benefits of incorporating NUS into agri-food systems to assist in solving existing regional food, nutrition, environmental and socio-economic challenges and contribute toward achieving the five pillars of the SDG agenda and specific sustainable development goals, particularly SDGS 1,2,3, and 15. The framework integrates the potential benefits of including NUS in agri-food systems to positive food, water, energy, environment, socio-economic and cultural systems, and human health outcomes. We connect these co-benefits to aiding the realization of SDGs and addressing food-nutrition-environmental challenges in the region. A need for transformation, especially within the policy and governance environment, to enable necessary changes in the agri-food systems is also highlighted in the framework.

## 5 Limitations

The synthesis was limited to research published between 2000 and June 2023. The study’s inclusion criteria were strict, and the search terms used were very specific; this may have inadvertently excluded some publications that would have been useful but were disqualified in the different screening phases. However, synonyms were used to avoid bias. The methodological quality of the studies included in the synthesis was not evaluated. The authors opted not to include gray literature publications due to the lack of verifiable and comprehensive information and publication language barriers, to name a few challenges. Access to appropriate gray literature would have broadened the scope of the synthesis on applying NUS. However, the authors chose to follow the quality-over-quantity approach to the literature included. This study does not include a comparative analysis of NUS and major crops. Rather it focuses on identifying pathways to mainstreaming NUS into the agri-food system and identifying the positive spinoffs from doing so. The authors collectively believe that the publications selected are a good representation of literature documented across different disciplines on the subject.

## 6 Conclusions and recommendations

In this review, we integrated existing research on the use and potential of underutilized crops to contribute toward solving recurring challenges in SSA. There has been a growing body of knowledge on NUS species over the past 23 years, particularly after 2015; however, their adoption is still suppressed by structural, institutional, socio-cultural and ideological barriers and lacking efforts to infiltrate them. Overlapping themes reported possible outcomes of NUS incorporation in agri-food systems, including health and wellbeing and environmental and socio-cultural outcomes. NUS have the potential to support food sovereignty and food and nutrition security by promoting culturally sensitive, inclusive, diverse farmer-driven agri-food systems and value chains where farmers, particularly women farmers, are not excluded in fundamental agri-food systems value chains. Bambara groundnut was the most researched and promoted NUS species due to its potential to contribute to various components of food and nutrition security in an environmentally safe way.

With a comprehensive review of 75 publications, we investigated the utilization of these crops, barriers hindering their utilization and an opportunity for their strategic positioning to inform the transformation of existing agri-food systems into sustainable, resilient, inclusive and diversified agri-food systems within safe planetary boundaries. What is common across the literature reviewed is that the current agri-food systems are outdated and unsustainable and are competing with local populations, and the environment for scarce resources such as water in SSA while failing to meet necessary requirements, particularly under climate crisis.

**Figure 10:**
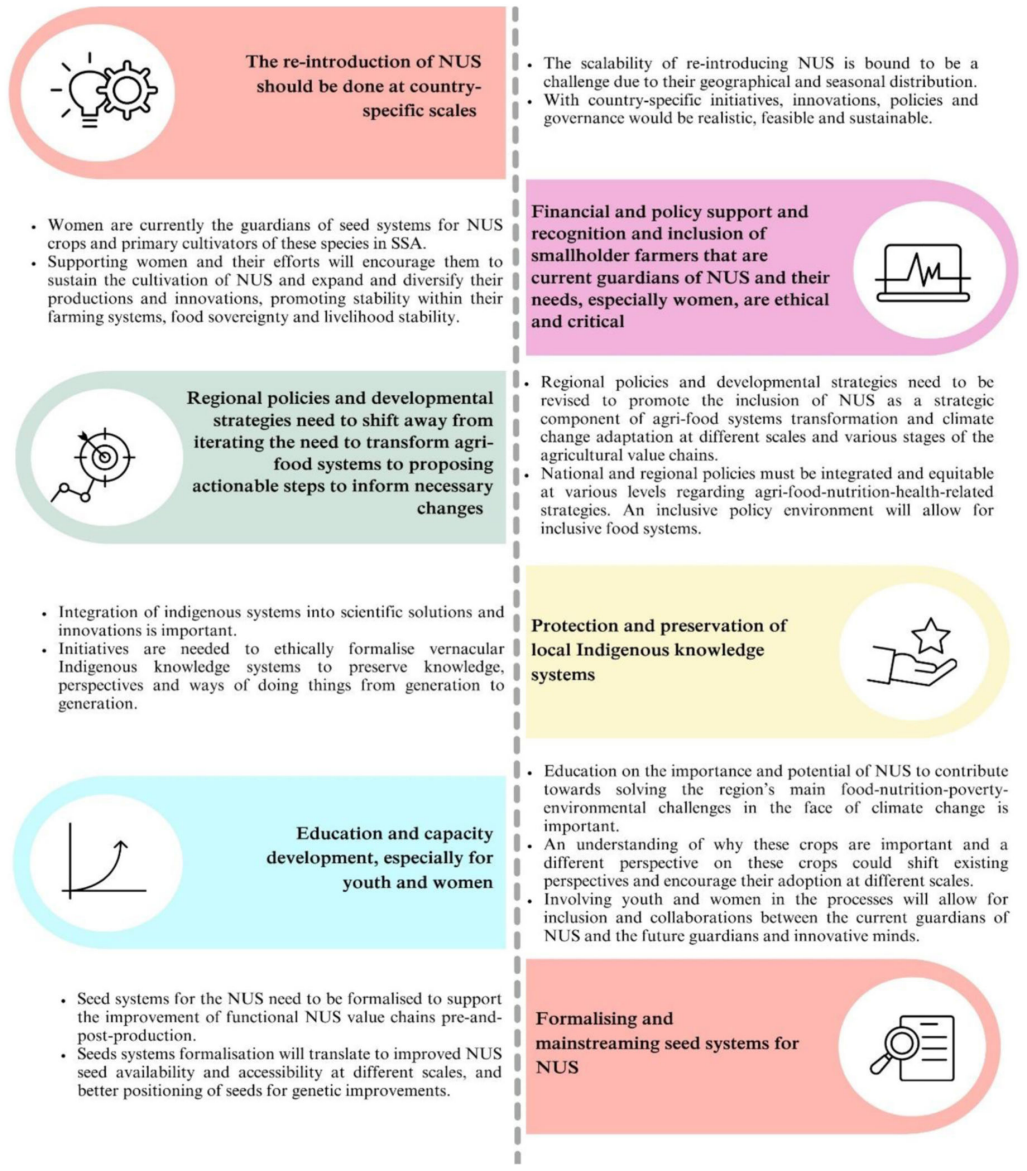
Study recommendations (presented in colored sections) and their explanations (in white sections). These are author recommendations based on the findings of the study.

The current mainstream agri-food systems are further among SSA’s prominent underlying causes of food and nutrition insecurity and poverty. NUS are promoted by research as a solution to transforming these agri-food systems. However, despite supporting these crops, currently implemented policies and agricultural development initiatives suppress NUS crops and are monopolized, reinforcing dependence on mainstream crops, exclusive, lacking contextualization, and not focusing on interventions sensitive to the environment and food and nutrition security. Transformation in the existing agri-food systems and supporting policies is necessary and urgent to address existing food, nutrition, environmental and socio-economic challenges and progress toward achieving Sustainable Development Goals, particularly SDGs 1(no poverty), 2 (zero hunger), 3 (good health and wellbeing), 5 (gender equity), 13 (climate action) and 15 (life on land) by 2030 in Sub-Saharan Africa. The evidence gathered in this review highlights a need for transformation across multiple sectors to solve existing challenges. Based on the evidence gathered in this review, the following recommendations apply for SSA (Figure 10).

## Supplementary Material

Supplementary Material

## Figures and Tables

**Table 1:** Study inclusion and exclusion criteria.

Inclusion criteria
- Full-text article available online in English and published in peer-reviewed journals
- Articles that have Indigenous (food) crops (and synonyms) as their primary subject
- Articles that have Indigenous (food) crops and climate change adaptation as subjects or climate change and Indigenous (food) crops
- Articles documenting the application of Indigenous crops and climate change in the SSA region
- Articles published between Jan 2000 and December 2023
Exclusion criteria
- Articles focusing on different geographical settings, outside SSA
- Articles that do not focus on Indigenous (food) crops
- Articles offered in other languages and not available in full-text and English
- Articles focusing only on chemical compositions, horticulture, crop-physiology, atmospheric sciences and or soil, crop and other environmental and climate change aspects and not the application of NUS

**Table 2:** Some socio-economic values of NUS crop species reported in the reviewed literature.

Socio-economic value	Explanation	Examples of published literature
-Enhancing local agri-food systems diversifies food sources and promotes food sovereignty and nutritional benefits	NUS are widely culturally acceptable and have a broader range of genetic variation. Promoting NUS as strategic crops will support the multi-stakeholder inclusion, agricultural diversity, resilience, and sustainability of local food systems. These crops could also be used to genetically enhance mainstream crops, resulting in increased resilience, adaptability and productivity of local agri-food systems.	Sidibé et al., 2020; van Zonneveld et al., 2021;Mabhaudhi et al., 2017b; Jiri et al., 2017; Agulanna, 2020; Cheng et al., 2017; Paliwal et al., 2020;Mbosso et al., 2020; Muthoni and Nyamongo, 2010; Hunter et al., 2019
-NUS requires fewer inputs and are easier to cultivate	NUS are highly adaptable and require fewer resources to grow. Promoting NUS to be mainstreamed into current agri-food systems could help communities achieve more stable production systems at lower production costs.	Mabhaudhi et al., 2017a; van Zonneveld et al., 2021; Bilali, 2020; Chivenge et al., 2015; Agulanna, 2020; Paliwal et al., 2020; Mbosso et al., 2020
-NUS can be buffer crops: avoiding hunger and poverty during lean seasons	NUS are better adapted to their local environments and changes to climatic and environmental conditions (Pironon et al., 2019). These crops, therefore, can serve as safety nets to protect communities from poverty and hunger during adversity.	Mabhaudhi et al., 2017a; Mugiyo et al., 2021;Akinola et al., 2020; Catarino et al., 2021; Momanyi et al., 2020; Koch et al., 2021; Pironon et al., 2019
-NUS are healthier alternatives	NUS are generally richer in healthy fats and carbohydrates, nutrients, micronutrients, minerals and proteins, which could help local communities bridge the existing nutritional gaps and sustainably achieve improved nutritional health.	Bilali, 2020; van Zonneveld et al., 2021; Momanyi et al., 2020; Hunter et al., 2019
-NUS can improve Gender inclusivity in agri-food systems, particularly under climate change	Women are the primary guardians of NUS and their seed systems in rural areas. Promoting these crops will enable women to participate as stakeholders in local agri-food systems and economies.	Agulanna, 2020; Catarino et al., 2021
-NUS inclusion can enhance economic opportunities and value chains for local people, especially women and youths	NUS crops aimed at specific niche markets at a local level have socio-economic and cultural potential. If local farmers are involved in such value chains, sustainable employment creation and autonomous pathways out of poverty could be created.	Mabhaudhi et al., 2016; Mashamaite et al., 2022; Adejumobi et al., 2022
-NUS can improve Local agri-food systems’ resilience, adaptability and stability	NUS are resilient and adaptable to changes in climate and environment, and they are, therefore, more stable in food systems compared to major crops. These crops, in this way, can ensure sustained and resilient production and agri-food systems value chains at different levels.	Nhamo et al., 2022; Chase et al., 2023; Akinola et al., 2020; Koch et al., 2021; Chemura et al., 2022; Morrow et al., 2023
-NUS can improve environmental health	Underutilized crops have been reported to have immense potential to rehabilitate, improve and strengthen local environments. Such environmental benefits include but are not limited to nitrogen fixation and improving environmental biodiversity.	Sidibé et al., 2020; Akinola et al., 2020; Catarino et al., 2021; Momanyi et al., 2020; Pironon et al., 2019

**Table 3:** Summary of drivers of adoption and adoption barriers of NUS. The drivers of adoption were conceptualized by authors based on the reported barriers.

Potential drivers of adoption	Reported barriers to adoption
Improved seed systems and seed availability through, for example, local stakeholder-inclusive seed banks	- Ill-defined seed systems and value chains (production, consumption, processing, feasible markets and product development) around NUS (Bilali, 2020; Hendre et al., 2019; Chivenge et al., 2015; Mabhaudhi et al., 2018b, 2016). - Perceived poor competitiveness of NUS in mainstream markets.
Building local community capacity and understanding of NUS benefits for food security, climate resilience, and shock absorption in local livelihoods	- Insufficient genetic and traditional data on NUS production, storage and processing (Sirany et al., 2022; Mekonnen et al., 2022; Mugambiwa, 2018; Boakye et al., 2018). - Ideological barriers and policies that are pro-mainstream crops pushing NUS to the risk of genetic erosion (Mabhaudhi et al., 2018b). Discouragement from incorporating such crops into farming systems due to institutional, socio-cultural and ideological barriers such as labeling them as “poor men’s crops” and neglecting and forbidding them in seed systems because they are “unregistered crop varieties.” - Poor and underdeveloped seed systems (primarily farmer-driven) (Mabhaudhi et al., 2016; Sidibé et al., 2020; Paliwal et al., 2020). - Long cooking time requires more resources such as wood and water (Mubaiwa et al., 2018).
Improving local agri-food systems, agrobiodiversity and dietary diversity in a sustainable and resilient way	- Most NUS are lower yielding compared to major crops (Sidibé et al., 2020). - Consumer preferences and reported attributes of some NUS crops, such as bad/bitter taste hindering utilization (Obidiegwu et al., 2020).
Their potential to reinstate food sovereignty	- Lack of relevant data and limited inclusion in developmental agendas as strategic crops. - Stereotypes and lack of information acknowledging the value of NUS (Hunter et al., 2019). - Neglect in genetic improvement efforts. - Lack of production data and planting knowledge and materials. - Lack of processing techniques and diversification of products of most NUS for consumption (Mubaiwa et al., 2018). - Lack of processing information, some NUS can be poisonous if not well-prepared.
Genetics-assisted breeding to help improve crop cultivars to require less processing time	- Lack of relevant genetic and phenotypic data on NUS.

## Data Availability

The original contributions presented in the study are included in the article/Supplementary material, further inquiries can be directed to the corresponding authors.
