# Supplementary material for: Underutilized crops for diverse, resilient and healthy agri-food systems: a systematic review of sub-Saharan Africa
Source: Front Sustain Food Syst. Author manuscript; Available in PMC 2025 Apr 24. (PMC7617609; doi:10.3389/fsufs.2024.1498402)
Supplement: Supplementary Material [file EMS204370-supplement-Supplementary_Material.DOCX]

# **Underutilized crops for diverse, resilient and healthy agri-food systems: A systematic review of sub-Saharan Africa**

**Supplementary Material**

**Text 1**

**WoS:**

**((TS=(Crop* NEAR/5 (indigenous OR neglected OR traditional OR orphan OR native OR underutili$ed OR future) ) AND TS=("climat* change" OR "climat* variability" OR "global warming" OR "extreme weather" OR "greenhouse effect*" OR (("carbon dioxide" OR co2 OR methane OR ch4 OR "nitrous oxide" OR n2o OR "nitric oxide" OR "nitrogen dioxide" OR nox OR "pm2.5" OR "black carbon" OR "organic carbon" OR "sulphur dioxide" OR "oxidized sulphur" OR "so2" OR "sox" OR "sulphuric acid" OR so4*) NEAR/5 (emit* OR emission OR releas* OR mitigat*) ) OR temperature* OR precipitat* OR rainfall OR "heat ind*" OR "extreme heat" OR "heat-wave" OR "heat wave" OR humidity OR drought* OR hydroclim* OR monsoon OR "el ni?o" OR enso OR SOI OR "sea surface temperature*" OR sst OR flood* OR storm* OR cyclone* OR hurricane* OR typhoon* OR "sea-level" OR wildfire* OR "wild fire*" OR "forest fire*" OR ( extreme* NEAR/5 event* ) OR "coast* erosion" OR "coastal change*" OR (disaster* NEAR/5 ( risk OR manag* OR natural) )) AND TS=("Sub-Saharan Africa" OR "SSA" OR "Sub Saharan Africa" OR "South Africa" OR "West-Africa" OR "West Africa" OR "East-Africa" OR "East Africa" OR "Angola" OR "Benin" OR "Botswana" OR "Burkina Faso" OR "Burundi" OR "Cameroon" OR "Cape Verde" OR "Central African Republic" OR "Chad" OR "Congo" OR "Djibouti" OR "Ivory Coast" OR "Cote d'Ivoire" OR "Eritrea" OR "Ethiopia" OR "Gabon" OR "Gambia" OR "Ghana" OR "Guinea" OR "Guinea-Bissau" OR "Kenya" OR "Lesotho" OR "Liberia" OR "Madagascar" OR "Malawi" OR "Mali" OR "Mauritania" OR "Mauritius" OR "Mozambique" OR "Namibia" OR "Niger" OR "Nigeria" OR "Rwanda" OR "Sao" OR "Senegal" OR "Seychelles" OR "Sierra Leone" OR "Somalia" OR "Sudan" OR "South-Sudan" OR "Tanzania" OR "Togo" OR "Uganda" OR "Zaire" OR "Zambia" OR "Zimbabwe" OR "Southern Africa" OR "East Africa" OR "West Africa" OR "Central Africa") NOT TS=(star OR "solar system" OR paleo*) ))**

**Scopus:**

**((Crop* W/2 (indigenous OR neglected OR traditional OR orphan OR native OR underutili?ed OR future)) AND ("climat* change" OR "climat* variability" OR "global warming" OR "extreme weather" OR "greenhouse effect*" OR (("carbon dioxide" OR co2 OR methane OR ch4 OR "nitrous oxide" OR n2o OR "nitric oxide" OR "nitrogen dioxide" OR nox OR "pm2.5" OR "black carbon" OR "organic carbon" OR "sulphur dioxide" OR "oxidized sulphur" OR "so2" OR "sox" OR "sulphuric acid" OR so4*) W/2 (emit* OR emission OR releas* OR mitigat*)) OR temperature* OR precipitat* OR rainfall OR "heat ind*" OR "extreme heat" OR "heat-wave" OR "heat wave" OR humidity OR drought* OR hydroclim* OR monsoon OR "el ni?o" OR enso OR SOI OR "sea surface temperature*" OR sst OR flood* OR storm* OR cyclone* OR hurricane* OR typhoon* OR "sea-level" OR wildfire* OR "wild fire*" OR "forest fire*" OR ( extreme* W/1 event* ) OR "coast* erosion" OR "coastal change*" OR (disaster* W/1 ( risk OR manag* OR natural))) AND ("Sub-Saharan Africa" OR "SSA" OR "Sub Saharan Africa" OR "South Africa" OR "West-Africa" OR "West Africa" OR "East-Africa" OR "East Africa" OR "Angola" OR "Benin" OR "Botswana" OR "Burkina Faso" OR "Burundi" OR "Cameroon" OR "Cape Verde" OR "Central African Republic" OR "Chad" OR "Congo" OR "Djibouti" OR "Ivory Coast" OR "Cote d'Ivoire" OR "Eritrea" OR "Ethiopia" OR "Gabon" OR "Gambia" OR "Ghana" OR "Guinea" OR "Guinea-Bissau" OR "Kenya" OR "Lesotho" OR "Liberia" OR "Madagascar" OR "Malawi" OR "Mali" OR "Mauritania" OR "Mauritius" OR "Mozambique" OR "Namibia" OR "Niger" OR "Nigeria" OR "Rwanda" OR "Sao" OR "Senegal" OR "Seychelles" OR "Sierra Leone" OR "Somalia" OR "Sudan" OR "South-Sudan" OR "Tanzania" OR "Togo" OR "Uganda" OR "Zaire" OR "Zambia" OR "Zimbabwe" OR "Southern Africa" OR "East Africa" OR "West Africa" OR "Central Africa") AND NOT(star OR "solar system" OR paleo*))**

**Which became too narrow for the engine and was simplified to accommodate a broader search to:**

**((Crop* W/2 (indigenous OR neglected OR traditional OR orphan OR native OR underutili?ed OR future)) AND ("Sub-Saharan Africa" OR "SSA" OR "Sub Saharan Africa" OR "South Africa" OR "West-Africa" OR "West Africa" OR "East-Africa" OR "East Africa" OR "Angola" OR "Benin" OR "Botswana" OR "Burkina Faso" OR "Burundi" OR "Cameroon" OR "Cape Verde" OR "Central African Republic" OR "Chad" OR "Congo" OR "Djibouti" OR "Ivory Coast" OR "Cote d'Ivoire" OR "Eritrea" OR "Ethiopia" OR "Gabon" OR "Gambia" OR "Ghana" OR "Guinea" OR "Guinea-Bissau" OR "Kenya" OR "Lesotho" OR "Liberia" OR "Madagascar" OR "Malawi" OR "Mali" OR "Mauritania" OR "Mauritius" OR "Mozambique" OR "Namibia" OR "Niger" OR "Nigeria" OR "Rwanda" OR "Sao" OR "Senegal" OR "Seychelles" OR "Sierra Leone" OR "Somalia" OR "Sudan" OR "South-Sudan" OR "Tanzania" OR "Togo" OR "Uganda" OR "Zaire" OR "Zambia" OR "Zimbabwe" OR "Southern Africa" OR "East Africa" OR "West Africa" OR "Central Africa"))**

**Table 1**

Data extraction table using Microsoft Excel

**Table 2**

**Appraisal Questions: Adapted from the Critical Appraisal Skills Programme (CASP) checklist**

| Is the study research question well formulated? | Yes | No | Unclear |
| --- | --- | --- | --- |
| Does the study have a clear methodology? | Yes | No | Unclear |
| Is the study synthesised and presented appropriately? | Yes | No | Unclear |
